# 3D Bioprinting in Limb Salvage Surgery

**DOI:** 10.3390/jfb15120383

**Published:** 2024-12-19

**Authors:** Iosif-Aliodor Timofticiuc, Serban Dragosloveanu, Ana Caruntu, Andreea-Elena Scheau, Ioana Anca Badarau, Nicolae Dragos Garofil, Andreea Cristiana Didilescu, Constantin Caruntu, Cristian Scheau

**Affiliations:** 1 Department of Physiology, The “Carol Davila” University of Medicine and Pharmacy, 050474 Bucharest, Romania; 2 Department of Orthopaedics and Traumatology, The “Carol Davila” University of Medicine and Pharmacy, 050474 Bucharest, Romania; 3 Department of Orthopaedics, “Foisor” Clinical Hospital of Orthopaedics, Traumatology and Osteoarticular TB, 021382 Bucharest, Romania; 4 Department of Oral and Maxillofacial Surgery, “Carol Davila” Central Military Emergency Hospital, 010825 Bucharest, Romania; 5 Department of Oral and Maxillofacial Surgery, Faculty of Dental Medicine, Titu Maiorescu University, 031593 Bucharest, Romania; 6 Department of Radiology and Medical Imaging, Fundeni Clinical Institute, 022328 Bucharest, Romania; 7 Department of General Surgery, The “Carol Davila” University of Medicine and Pharmacy, 050474 Bucharest, Romania; 8 Department of General Surgery, “Dr. Carol Davila” Clinical Hospital of Nephrology, 010731 Bucharest, Romania; 9 Department of Embryology and Microbiology, Faculty of Dentistry, The “Carol Davila” University of Medicine and Pharmacy, 050474 Bucharest, Romania; 10 Department of Dermatology, “Prof. N.C. Paulescu” National Institute of Diabetes, Nutrition and Metabolic Diseases, 011233 Bucharest, Romania; 11 Department of Radiology and Medical Imaging, “Foisor” Clinical Hospital of Orthopaedics, Traumatology and Osteoarticular TB, 021382 Bucharest, Romania

**Keywords:** 3D bioprinting, limb salvage surgery, biocompatible, prostheses, electron beam melting

## Abstract

With the development of 3D bioprinting and the creation of innovative biocompatible materials, several new approaches have brought advantages to patients and surgical teams. Increasingly more bone defects are now treated using 3D-bioprinted prostheses and implementing new solutions relies on the ability of engineers and medical teams to identify methods of anchoring 3D-printed prostheses and to reveal the potential influence of bioactive materials on surrounding tissues. In this paper, we described why limb salvage surgery based on 3D bioprinting is a reliable and effective alternative to amputations, and why this approach is considered the new standard in modern medicine. The preliminary results of 3D bioprinting in one of the most challenging fields in surgery are promising for the future of machine-based medicine, but also for the possibility of replacing various parts from the human body with bioactive-based constructs. In addition, besides the materials and constructs that are already tested and applied in the human body, we also reviewed bioactive materials undergoing in vitro or in vivo testing with great potential for human applications in the near future. Also, we explored the recent advancements in clinically available 3D-bioprinted constructs and their relevance in this field.

## 1. Introduction

**Figure 1:**
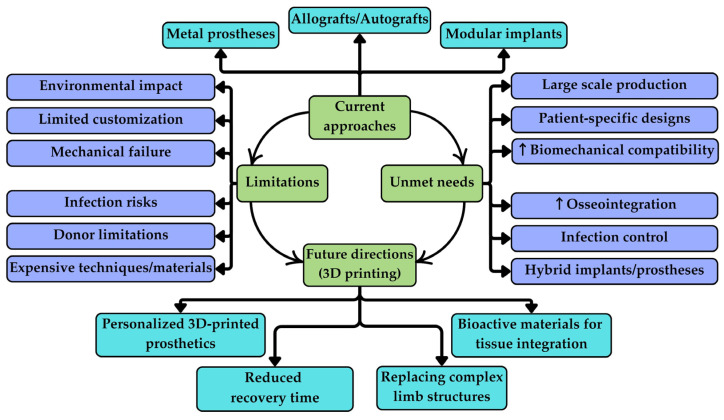
Roadmap of limb salvage surgery approaches, limitations, unmet needs, and the perspectives of 3D printing in improved personalized patient care. ↑ = increased/enhanced.

In the last decade, medical and bioengineering teams aimed to find a better solution for salvaging limbs affected by various diseases or trauma while avoiding amputation. As presented in a study on lower-limb amputations, approximately 79% of the individuals who underwent amputations suffered from a vascular disease [1]. Pathology of the vessels may affect the surrounding tissues, inducing inflammation and oxidative stress, and reducing the protective mechanisms of the surrounding cells [2]. In severe cases of extensive destruction of muscles, bones, connective tissue, nerves, arteries, and veins, the classical amputation remains the optimal approach unless one or more of these components can be replaced. Moreover, various traumas of the limbs or some systemic diseases such as malignancies lead to limb amputations [3,4]. With the fast development of 3D printing, followed by the emergence of a new branch-3D bioprinting, as well as a parallel advancement in biocompatible materials, the distance has shortened to the point where the need for amputations can be replaced with saving limbs. Limb salvage surgery is an innovative approach in medicine that appeared with the emerging field of microvascular surgery around the 1960s, and it has flourished due to the development of different 3D bioprinting techniques and the possibility to create unique and functional builds [5]. As long as a revascularization of the affected area is possible and a reconstruction or a replacement of the affected body part can be performed, the necessity for amputation is very low to none [5]. In addition, limb salvage surgery aims to reduce the high mortality associated with amputations [6]. Standard limb salvage surgery is very effective in maintaining the function and quality of life; however, it faces several unmet clinical needs. Most importantly, traditional approaches often rely on modular prostheses, allografts, or metallic implants, which may not be adequate for complex bone defects. This may lead to poor implant fit, limited integration with host tissues, or higher risk of complications such as infection, implant loosening, or mechanical failure [7,8,9]. Additionally, conventional materials are not able to mimic the biomechanical and bioactive properties of natural bone, leading to longer recovery times and worse functional outcomes [10,11]. Moreover, for the majority of patients who suffer from vascular disease, a solution for optimal revascularization to perform such salvaging surgeries has not yet been identified. However, there are reported studies about 3D bioprinting vessels, so once the optimal balance between viable cells and other biomaterials that offer integrity to the blood vessels is found, this challenge could be approached [12,13]. Also, if two or more different tissues of the limb are affected, salvage surgery is not a solution due to the difficulty of predicting how two different scaffolds of materials with different purposes interact with each other [5] (Figure 1).

In recent studies on 3D bioprinting, some researchers define the technique as the use of 3D printing for building three-dimensional components for use in the human body or for medical research or simply tissue engineering. However, more rigorous studies consider 3D bioprinting as the field where 3D printing techniques are used to print with viable cells or bioinks [14,15]. Yet, from our point of view, a more accurate definition of 3D bioprinting would be of a field that emerged as a 3D-printing medical branch that aims to use viable cells and nonviable materials or mixes between the two to build a construct to be used in the human body from a functional or strictly structural perspective while also including constructs such as tissue engineering scaffolds that support the development of treatments and new medical or surgical approaches [15,16,17,18]. However, to avoid confusion and keep the rigor of the studies, for the purpose of this paper we refer to bioprinting as a 3D-printed construct that includes viable cells.

Following the emerging field of microvascular surgery, the development of limb salvage surgery was supported by new approaches based on constructs created using 3D-bioprinting technologies, such as entire bones, ligaments, and muscles with a key part represented by the properties that these biomaterials show, such as angiogenesis and osteogenesis [19].

In contemporary vision, the only solution to rebuild an entire limb is using 3D-printed biomaterials. As long as an optimal balance is found between the bioactive properties of different materials, we can expect great advancements in the future of regenerative medicine, where in extreme cases, entire limbs are 3D printed and attached to the body in functional harmony.

The purpose of our paper is to highlight the recent approaches and innovations in 3D bioprinting applicable to limb salvage surgery. However, many of the studies that approach 3D printing in limb salvage surgery do not focus on the interaction between the prostheses with the rest of the native tissues. Therefore, considering the limited number of studies found regarding the bioactive properties of the materials used in bioprinting and 3D printing limb parts, we believe that any approach that considers the biological activity and functionality of the builds is innovative in limb salvage surgery. We addressed the current state of development of the field, ranging from recent discoveries and in vitro tests on compounds with potential in bone regeneration and growth to research on animal models that offered solutions for the future of 3D bioprinting in limb salvage surgery. Also, we comprehensively reviewed the recent advancements in clinically available 3D-bioprinted constructions and the promising results of these studies for patients and surgical teams tackling major limb surgery.

## 2. 3D Bioprinting in Orthopedics

The increasing interest in the emerging field of 3D printing is doubled by the necessity for new approaches in limb salvage surgery. As such, extensive research is performed in this area to find solutions for patients with bone defects that normally require amputations [20]. Initially, the main approach was to identify designs and materials that mimic the mechanical behavior of the bone; therefore, metal prostheses made of titanium or tantalum were designed as a new solution for limb salvage surgeons [21,22]. However, with the rapid development of this field, the aim went beyond fulfilling the mechanical characteristics of the bone. An innovation in bone 3D printing is creating a 3D-printed build that can behave as a physiological bone, an active tissue that renews itself, interacts with the surrounding tissues, and influences the homeostasis of the body [23]. To aid in this problem, many biocompatible materials were designed to behave as active tissue in light of their bioactive properties, capacity to promote the activation of the surrounding cells, and limited effect in generating inflammation or being harmful to the body in the long term [24].

### 2.1. In Vitro Studies

Through in vitro testing of different materials, new compounds were identified or designed that can closely mimic the mechanical behavior and bioactivity of the bone, promoting osteogenesis, angiogenesis, and proliferation of the adjacent tissues [25,26].

Only a small fraction of the biomaterials tested on animal models can be clinically implemented; therefore, metal prostheses still represent a reliable solution in orthopedics [27]. However, even though many materials are still in the phase of in vitro testing, they represent a promising solution for the future of 3D bioprinting in limb salvage surgery in light of the recent and innovative results regarding their bioactivity. After the rapid implementation of the classical 3D-printed limb parts in the field of limb salvage surgery, with the development of 3D bioprinting, the main focus switched to finding optimal materials that can promote cell differentiation, thus ensuring an enhanced integration of the prostheses created, in patients [28].

Silk fibroin (SF), a natural protein extracted from silkworms, was tested in vitro and in vivo to assess its potential effects on bony tissue [29,30]. It was concluded that SF presents heightened biocompatibility and does not produce immunological responses when transferred in vivo. In vitro, testing showed low cytotoxicity along with bioresorbability and biodegradability, properties that are mandatory for a material that is required to mimic the bone tissue [31]. The similarities with bone tissue functionality and structure are not sufficient to create a construct that presents unique characteristics for specific patients’ needs, but in our opinion, a material needs to present a minimum of bioactive properties to be a potential candidate for bioprinting and for future integration with other scaffolds or native tissues.

Even though many forms of SF were created, such as films, hydrogels, and even nanoparticles, that can be 3D-printed in different scaffolds with bioactive properties and great mechanical resistance (strength of 300–740 MPa and toughness similar to the cortical bone) [31,32], it was also shown that SF can induce the differentiation of human mesenchymal stem cells (MSCs) and promote VEGF synthesis. Thus, SF represents a material optimal for the induction of bone cell proliferation and regeneration while also promoting angiogenesis [33]. Interpreting these results, we can conclude that SF has great compatibility with human cells, which means that integrating any other viable cells with SF in bioinks is an optimistic possibility for the future of bioprinting in limb salvage surgery.

Similar bioactivity with SF can be observed in natural polysaccharides, such as alginates, that were also used in building scaffolds mimicking bone activity. Alginates are substances that can be 3D printed as bioinks, and stem cells can therefore be added to these compounds for optimal promotion of cell growth and low cytotoxicity [34]. Not different from SF, alginates promote the differentiation of MSC and can perform as well as cortical bone in mixtures with bone morphogenetic proteins. However, due to the lack of mechanical properties of alginates, building a prosthesis showcasing alginate’s properties requires methods of integrating these polysaccharides with metal or other rigid material structures [35]. These drawbacks of integrating soft materials in 3D-bioprinted limb parts can be frequently observed throughout the studies mentioned in this review. For now, an accessible solution to this challenge is to create hybrid builds. A solution could be 3D printing solid parts, to offer integrability and mechanical properties to the constructs, and then coating them with various classical 3D-printed bioactive coats or applying bio-printed scaffolds to the surfaces or inside the structure.

Many materials, especially biopolymers such as PLLA (Poly-L-Lactic Acid), PCL (Polycaprolactone), PLA (Polylactic Acid), PEEK (Polyetheretherketone), HA (Hydroxyapatite), and β-TCP (β-Tricalcium Phosphate) present bioactive properties in the range of cell proliferation, osteoblast proliferation, osteoinductivity, osteoconductivity, and even angiogenesis [24]. However, various in vitro activities can be stimulated in different compounds; for instance, collagen was observed to deliver proportionally different substances in the surroundings such as active compounds or antibiotics. This property can be classified as a 4D activity, i.e., during the slow degradation in time, the material gradually releases the substances with which it was loaded [36].

Bone morphogenetic proteins are clinically approved and used as loads in 3D-printing constructs to promote bone cell proliferation. In a recent study, a replacement for this classic method of osteoblast induction was researched on ST2 bone marrow-derived cells placed in a scaffold made of PCL and GelMA in which a few nanograms of Wnt3a (osteoinductive protein) were included. It was shown that in the areas where this protein was added, the quantity of alkaline phosphate was high, a marker showing osteoblast differentiation. This promising result could represent a future possibility of treating small bone defects or even usage for healing critical bone defects [37]. Considering these promising results, we believe scaffolds or implants based on this composition can also be created and applied in patients with less severe cases of bone trauma, especially in those who do not necessarily need limb salvage surgery.

Considering that the majority of innovative compounds that can now be 3D printed are biopolymers [38], those with great structural integrity are useful when porosity areas are vast. A relevant example is hydroxyapatite, which presents enhanced metabolic activity and induction of osteoblast differentiation.

However, it is the responsibility of the research teams to find optimal balances in mixtures that may represent a concrete solution in limb salvage surgery and other clinical applications.

### 2.2. In Vivo Animal Models

Total or partial bone replacement in animal models represents a great solution for the transition to the implementation of these 3D-printed prostheses in humans. Alongside the in vitro tests conducted on these materials, animal models allow for the observation of the interaction of the material with the surrounding tissue [39]. This encourages research for methods to fixate the prostheses in the affected limb. Thus, a smoother transition can be made to clinical practice, especially with prostheses designed from already approved and biocompatible materials.

There is paucity of clinical information regarding the interactions of the patient’s surrounding tissue with the prostheses. In this issue, a research team performed tests on animal models for radius/ulna-scaffold complexes with biologically active coatings composed of substances such as dipyridamole used as an enhancer for bone formations or collagen [40].

Unique in this radius/ulna scaffold is the use of calcium phosphate-based alloplastic materials, which are known as one of the best bone substitutes, but with challenges in 3D printing due to their fragility and underperforming mechanical properties [24]. In a rabbit model, it was concluded that β-Tricalcium Phosphate scaffolds coated with high concentrations of dipyridamole (1000 µM) very precisely mimic the compact bone [40].

In another animal model testing of ulnar bone replacement, a 3D-printed PLLA model was built with an active and osteogenic compound, bone morphogenetic protein 2 (BMP2) for enhancing bone regeneration [41]. In addition, the PLLA scaffold was also coated with an alginate catechol and collagen to ensure optimal speed and regulation in the release kinetics of the osteogenic compound for 2 months, enough to facilitate the full incorporation and adaptation of the prosthesis in the animal model [41].

Regarding the lower limb, femoral defects may be one of the most extensively researched topics regarding 3D printing solutions, with a major focus on solutions for promoting osteogenesis and angiogenesis [42]. While metal prostheses still represent the majority, the use of polymer-based prostheses is increasing [43]. As already mentioned, polymers are similar when discussing the mechanical properties and superior in terms of the bioactivity of the material in the human body [24].

Another future perspective in medical 3D printing is the attempt to build polymeric prostheses with different compounds that can be added in the process of 3D printing, such as cell-proliferation-inducing substances or even stem/stem-based cells, thus creating bioinks. However, these solutions are underdeveloped for patient applications, and not all test substances have been approved as safe.

A research team tested the functionality of a PCL scaffold, 3D printed with FDM technology in a rat model of a femoral defect, in which stromal-derived factor 1 was added [44]. It was concluded that PCL combined with this bioactive compound was essential for optimal induction of osteogenesis, which led to the perfect healing of the femur. In addition, no cytotoxicity or other side effects were reported [44]. Many other combinations of substances such as PCL and β-TCP, PCL/Nanoparticulate Willemite composite, PCL/hydroxyapatite, porous tantalum with polydopamine, or quaternized chitosan-grafted polylactide-co-glycolide/hydroxyapatite were reported for the induction of osteogenesis for healing large bone defects, and the best mixture is yet to be identified; however, continued testing on the animal models is needed for the future transfer and application of these methods to humans [45,46,47,48]. Besides trying to integrate these results into the human surgery field, introducing human viable cells should also be considered. Observing the low cytotoxicity of the biopolymers, viable cells could not only resist inside the 3D-bioprinted scaffolds, but could also potentiate the mentioned bioactive properties.

Tumoral pathology has a serious and damaging evolution when occurring in the limbs, and in more severe cases may affect multiple compartments to a various extents [49,50,51,52]. These cases are frequently the subject of limb salvage surgery, and solutions are presently researched on animal models. These include the development of prostheses that fulfill the mechanical roles of the affected bones while behaving as real, bioactive tissue, in continuous communication with the adjacent structures. Substances such as cerium oxide, hydrogels, stem cells BMP-2 and hMSC, and natural collagen were introduced in polymer-based prostheses (PCL, PEEK, HA) for the promotion of proliferation and differentiation of osteoblasts and angiogenesis. They have shown promising results in terms of possible future application in human orthopedics and are expected to be perfected to become the next gold standard in limb salvage surgeries [53,54,55,56,57,58,59].

### 2.3. In Vivo—Clinically Available Approaches

Three-dimensional bioprinting emerged as a novel and innovative branch of the 3D-printing industry and was rapidly developed with the desire to obtain patient-specific treatments with near to no inflammatory response and rapid healing with fewer complications. The first materials used as scaffolds in 3D bioprinting were rigid and exhibited suboptimal mechanical properties, and this could be one of the reasons why orthopedics was a suitable medical sector for the initiation of bioprinting and its development [60].

Metal-based 3D-printed prostheses represent the vast majority of the clinically approved and applied constructs in today’s surgery practice. Polymer-based 3D-printed prostheses (or non-metal prostheses) represent a good future perspective for limb salvage surgery, with significant research still in progress, and a series of materials to be approved for use in the human body. The advent of digital 2D and especially 3D planning has made it possible to create excellent fitting of patient-specific implants concerning the recipient anatomy [61].

As mentioned in the introduction, some studies define bioprinting as 3D printing with materials that incorporate viable cells. In the case of bones, the data regarding 3D printing with mixes that contain viable cells are scarce. However, extensive research can be found about 3D-printed builds that mimic the biological and mechanical properties of the bone tissue.

#### 2.3.1. Metal-Based 3D-Bioprinted Constructs

Defects in the bones of the upper limb represent a great challenge for patients due to function deprivation, and it is the role of medical teams to support fast patient recovery while also considering aesthetics as well as the full recovery of all the functions that were lost due to trauma, infections, tumor growth, or other diseases.

The clavicle is the most frequently fractured bone of the scapular girdle, with a reported incidence of up to 5% of clinically presented fractures in adults [62,63]. According to the Orthopaedic Trauma Association and Canada Orthopedic Trauma Society, approximately 80% of clavicle fractures are located in the midshaft of the bone [64,65]. While some of these fractures may be treated by immobilization alone, many clavicle fractures cannot be healed without surgical intervention, which often implies fixation with plates [66], requiring intraoperative manual adjustments. In the early stages of 3D printing development for orthopedic applications, a large number of prints were used for building bone models for accurate preoperative patient-specific prostheses or plate adjustments. It was concluded that using these new 3D printing technologies was helpful for the surgical teams by way of reducing the incision dimensions and time of the operation [67], especially for patients who reported less discomfort, since it resulted in fewer interventions for implant removals [68]. Three-dimansional printing is a great solution for limb salvage, as shown in the case of a 21-year-old female patient with clavicle Ewing’s sarcoma, where a 3D-printed titanium clavicle was manufactured for total bone replacement. Even though it is known that titanium is not an ideal material, it can lead to great results and can save patients from amputations [69]. Titanium can easily achieve the mechanical requirements for total bone replacement. While using metal-based prostheses replaced the need of amputation in patients, metal-based constructs could be coated or integrated with bioprinted scaffold to promote osteogenesis and angiogenesis for better integration of the entire bone with the surrounding tissues [70]. Furthermore, it can also be loaded with antibiotics to reduce the risk of infections or with human viable cells that present great characteristics in vitro but were not yet approved for clinical use, such as ST2 bone marrow cells [37,71].

Scapular bone fractures are infrequently compared with clavicular bones, representing under 5% of shoulder girdle fractures and insignificant among all clinically presented adult fractures [72]. However, in a few cases of the malignancies present in the shoulder girdle area, the scapula may be affected [73]. The most common type of bone sarcoma in adults is chondrosarcoma; however, Ewing’s sarcoma can also represent a significant percentage of scapular sarcomas, presenting a great risk of undergoing amputation or total removal of the bone [69,74].

**Figure 2:**
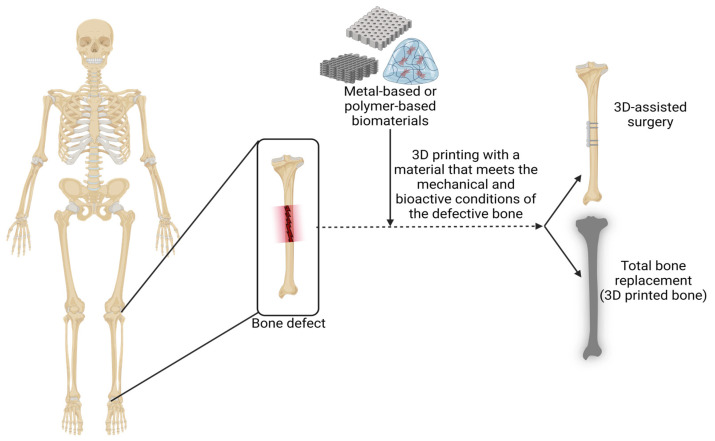
Three-dimensional printing constructs can serve as accessories in various orthopedic surgeries or as the main components in limb salvage surgery where 3D bioprinting is used to create entire bone prostheses. Created in BioRender. Timofticiuc, I. (2024) BioRender.com/w87n517, date of last access—12 October 2024.

In extreme cases of scapular bone disease, solutions from the 3D bioprinting area are under development for the creation of biological active prostheses that can spare the patients from the inflammatory rejections or the discomfort generated by more classical approaches. Some other defects that the scapular bone can suffer from, such as scapular bone fractures and scapular aneurysmal bone cysts, have already been resolved using 3D-printing instrumentations or medical accessories that assist the surgical teams in a patient-specific treatment; this leads to a reduction in intraoperative time and complications, as shown in Figure 2 [75,76,77,78].

In the case of a 35-year-old woman diagnosed with right scapular Ewing’s sarcoma [69], a technical 3D-printed solution was found, showcasing the vast possibilities of molding the titanium powder in different shapes and sizes. In this manner, the built prostheses do not have any specific requirements to be suitable for 3D printing; however, some drawbacks can appear when designing fixation methods for preserving the majority of the functions of the removed bone.

In a case study of four patients presenting with chondrosarcoma, it was determined that they required partial scapulectomy and reconstruction of the glenoid fossa, the neck of the scapula, and the humeral head. In this regard, a solution using 3D-bioprinted builds made of titanium alloy of the glenoid prosthesis and reverse shoulder prosthesis was identified, resulting in a short-term follow-up with no complications, no advancements of the malignancy, and great mobility recovery [79]. This success revealed the rapid and efficient evolution of the patients when they are treated using 3D-printing methods over a traditional approach, which may result in implant rejection or, in more severe cases, limb amputation.

One of the most serious challenges in orthopedics remains total or partial replacements of the bones affected by tumors or infections. Both primary and metastatic lesions may significantly affect the structural integrity of the bone and expand to adjacent soft tissue structures [80,81,82,83]. As mentioned, 3D bioprinting offers promising initial results [84,85,86,87,88,89,90,91,92,93,94]. In the case of a 5-year-old female patient presenting with advanced osteosarcoma of the proximal humerus, partial removal of the bone was necessary. The early age of the patient represented a challenge and a solution was found by building a 3D-printed titanium alloy prosthesis to replace the affected part of the bone. In this particular groundbreaking case, after a 3-year follow-up, the patient was tumor-free, pain-free, and had partial functionality of the arm. These results are very encouraging for the pediatric orthopedic and oncologic sectors, demonstrating that alternative treatments can be found to avoid amputation, providing great contribution to limb rescue operations [95]. Given the cause of the trauma in this patient, future approaches should also include bioprinting these prostheses with viable cells that promote controlled cell differentiation.

Compared to patients under somatic development, adult patients present fewer challenges and more compliance for postoperative medical recovery; also, they require fewer revisions for adjusting the prosthesis size to the dimensions of the bone [96,97]. Relatively few cases were reported for total elbow replacement; however, in the case of a 60-year-old patient who lost the range of motion in the elbow due to distal humerus fractures, a solution with a prosthesis made of titanium alloy was found, and after the surgery and a few weeks of recovery the patient regained initial functionality, showing once again the promising results of 3D-printed prostheses in ensuring the mobility of the limbs or in more serious cases, even saving them [98]. For elbow replacement, salvage surgery might resort to the initial use of megaprotheses; however, this may imply difficult later revisions and possible limited functionality, so fitting a patient-specific 3D-printed implant is ideal in this category of patients [99].

Forearm bone defects can have multiple causes, and similar to previous examples, these defects can be caused by trauma resulting in fractures that may require surgical treatment [100]. In forearm defects caused by infections, traumas, sarcomas, or hereditary conditions, one challenge is restoring the mechanical behavior that the ulna and radius ensure; therefore, 3D bioprinting shows good potential in the functional recovery of the elbow and the wrist after replacements with total or partial prostheses. An easy solution to approach small bone defects is by bioprinting scaffolds made of silk bioinks and alginate bioinks that promote osteogenesis [101,102]. Furthermore, combining the builds with antibiotic coatings or nanoparticles coating could help reduce the infections that initially caused the bone trauma [103].

Due to a leiomyosarcoma of the radius, a 53-year-old man underwent total resection of the radius along with the radial nerve, and a 3D-printed biological prosthesis was designed and built for replacement based on CT scans of the healthy radius [95]. The titanium alloy prosthesis of the entire radial bone allowed the patient to regain basic functionality after 3 years, despite the postoperatively dropped hand caused by the resection of the radial nerve.

One of the most complex biomechanical behaviors of the human body is represented by hand movements [104]. Thus, 3D printing hand components represents a greater challenge than other long-bone prostheses because of the need to maintain the functional and aesthetic purposes of the segment. Several applications in hand surgery using 3D printing were reported. Designing plates for fracture treatment as well as surgical guides are some examples, and they were employed using methods adapted from procedures on larger and longer bones [105,106,107,108,109,110,111,112,113].

Alongside fractures, a greater risk of hand-associated diseases and defects is represented by giant cell tumors, which can often lead to infection or amputation [114,115]. The treatment of giant cell tumors is complex, with numerous therapeutic options; however, production costs and the multitude of recurrent cases represent a significant drawback in the implementation of 3D-printed approaches. Tissue engineering, especially the 3D-bioprinting branch, provides solutions not only for the production cost and time of design, but also for the lack of highly experienced prostheses specialists.

In 2 years, seven patients presented with scaphoid and lunate fractures, and due to osteonecrosis of the carpal bones, total bone replacement was mandatory. There are few reported studies using titanium alloys as biocompatible materials for bony structures dated before 2020 [116,117]; however, in this particular study, the prostheses were built using porous tantalum, a metal considered to have better mechanical properties than titanium and more efficient integration in the patient’s limb [118]. As mentioned later in this article, porous surfaces promote an enhanced interaction of the 3D-printed build with the surrounding cells and proteins. Creating a hybrid construct by adding a coating over metal prostheses that presents porous surfaces but also bioactive properties would therefore greatly increase the functionality of these prostheses in patients.

There is a trend in advancing from metal prostheses to polymer prostheses. However, in the case of metacarpals, the literature on metal prostheses is scarce. In the case of three patients presenting with metacarpal giant cell tumors, total bone removal was mandatory [119]. Titanium alloys known for their mechanical strength and biocompatibility were used in building metacarpal prostheses for these patients, and a satisfactory recovery was reached [119]. Similarly, in the case of a 64-year-old man with a giant cell tumor of the proximal phalanx of the fourth finger, a 3D-printed titanium prosthesis was designed in an attempt to salvage the finger after the patient refused amputation [120].

The bones of the upper and lower limb do not present significant differences in morphology, and pathologies such as tumors, infections, or fractures are similar in terms of pathogenesis [121,122,123,124]. However, the biomechanics of the lower body bony structures are different due to the requirement of sustaining the body weight; therefore, resistance, support, and strength of the lower limbs overcome the mobility and complexity of the functions of the upper limbs [125,126]. The bones of the lower limb are also subjected to load-bearing-related deformity, and specific surgical corrective procedures with adequate materials and techniques may be required [127,128]. Restoration of alignment plays a major role in functional recovery and sustained load-bearing leads to better outcomes in terms of implant integration and bone quality [129,130].

However, femoral defects can produce extensive lesions which frequently lead to unwanted limb immobilization associated with severe pain [131,132]. Due to the novel 3D-bioprinting techniques developed in the last decade, such as extrusion-based 3D bioprinting, inkjet-based 3D bioprinting, laser-assisted 3D bioprinting, and stereolithographic-based 3D bioprinting [133], more solutions are available for patients, ensuring a partial or even total rendering of certain functions of the limb. While osteosarcoma is a serious disease with a high risk of metastasis, significant issues are also present in the case of giant cell tumors and even fractures, which often require total or partial replacement of the affected bone [131,132]. In a comparative study of 10 patients with giant cell tumors of the distal femur, the differences in disease evolution and healing were observed. Five patients were treated with cement packing with extra fixation reconstruction of the distal femur while the other five patients were treated using an innovative 3D-bioprinting method [134]. It was concluded that the 3D-printed strut-type prostheses made from titanium alloy in different models performed better in terms of mechanical stress on the knee joint. In addition, the capacity of the prosthesis is superior to cement reconstruction due to the osteoinduction and cell proliferation with curative effect. An innovation in this field could be represented by enhancing these properties of porous metals through integrating the builds with bioprinted scaffolds. Imitating the cancellous bone structure at the site of the distal femur, the titanium 3D-printed prostheses did not promote the disease recurrence nor metastases, despite their effects on cell proliferation [134]. The post-operative knee joint alignment is essential in restoring functionality but is also related to mechanical stress and wear of the metallic components and their effects on adjacent bone with physiological weight distribution [135]. Tibial and fibular defects have a variety of solutions ranging from surgically inserted plates and bone grafts to even tissue engineering. However, several drawbacks can appear due to the mechanical stress occurring in the adjacent joints of these bones. Through different tissue engineering solutions made available by 3D bioprinting, faster functional recovery and improved healing were obtained while also decreasing the number of needed revisions [136].

In a retrospective study on 14 patients reporting on the implantation of titanium alloy 3D-printed prostheses, the effectiveness and compatibility for the reconstruction of the tibial diaphyseal critical defects caused by osteomyelitis were assessed [137]. Although numerous methods are reported for the treatment of defects larger than 10 cm, it was concluded that besides four patients that needed a second intervention due to fixation screw failure, this method is optimal for the reconstruction of the tibial bone; it also appears very promising taking into consideration the possible addition of various bioactive substances [137].

In the case of the fibula, the only applications available in the literature regarding 3D printing are fracture plates and additional instruments used for fibular bone fracture. However, in any case of an entire tibial replacement, we need to consider the interaction of the prosthesis with the fibula and identify ways of fixating it in the surrounding muscles and cartilage but also to the fibula. In a reported study about tenogenic differentiation, it was shown that MSCs can differentiate into tendon cells [138]. As previously shown, there are reported studies where MSCs are bioprinted as scaffolds. Therefore, a solution for the fixation of muscles to the fibula is creation of a bioprinted tendon that promotes cell differentiation and could easier attach the muscles to the bone.

**Figure 3:**
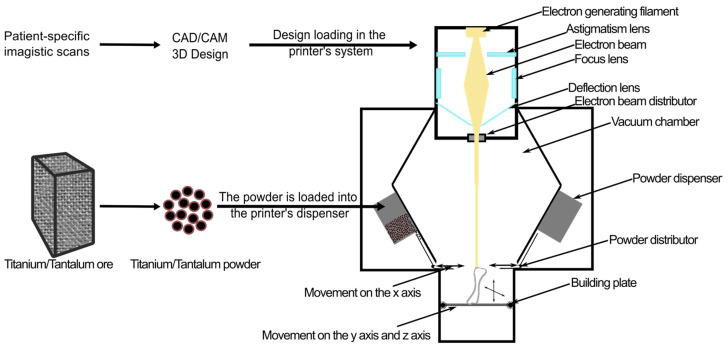
Electron Beam Melting (EBM) is the most frequently used technology for 3D printing metals such as titanium or tantalum. After the metal is processed in powder form, a special component distributes the powder as a thin layer at the top of the construction where an electron beam melts it, forming a new layer according to CAD software instructions.

In Figure 3, a visualization of the process of 3D printing using metals can be observed. In the application of metal-based prostheses, the possibility that they could pierce the bone or the surrounding tissues needs to be mentioned. Even though these products perform very well and do not easily degrade or break under mechanical stress, this possibility always exists as a potential complication, with increasing chances in case of great efforts or sudden movements [139].

#### 2.3.2. Non-Metal-Based 3D-Bioprinted Constructions

PEEK (polyether–ether–ketone) is a biodegradable material that exhibits great physical and mechanical properties, such as low Young’s modulus [140]. Its introduction encouraged studies and development of prostheses models and it was rapidly concluded that it could present better results than titanium constructs. Even though it is not yet fully clinically approved, PEEK could represent the next ideal material with medical applications, especially in orthopedics [140].

In the case of a 23-year-old patient who presented with chronic right clavicle osteomyelitis, a 3D-printed clavicle was the solution. The part was accurately 3D-printed using CT scans and designed for fixation in the acromial end of the clavicle and the sternal stalk using PEEK. This provided optimal resistance of the prosthesis to the mechanical stress in the scapular girdle by reducing the stress-shielding effect while also ensuring no inflammatory responses and stimulating of osteogenesis [24,141].

It is interesting to note that bones not subjected to high mechanical stress as those in the shoulder girdle are replaced by 3D-printed prostheses made of materials with weaker mechanical results than titanium or PEEK but which present properties closer to the bone as well as enhanced bioactive and osteogenic capacities, such as hydroxyapatite, β-TCP, or PLLA.

PEEK presents numerous qualities and functionality when used in the construction of a prosthesis [24], and in the past few years, research teams are striving to develop and use polymers instead of metals. Scaphoid prostheses were designed and 3D-printed using a PEEK medical filament to demonstrate the effectiveness of the printing from a financial point of view and to showcase the ease with which a construct can be built specifically to the patient’s needs [142].

The bioactivity problem of the titanium alloy in tibial defects was addressed in a study using additional bioceramic granules made from β-TCP for osteoinductive activity promotion and enhanced metabolic activity [143]. In these cases, patients aged 13 or younger who presented with an osteosarcoma-induced defect in the tibial bone were subjected to bone replacement with biocompatible and bioactive titanium-bioceramic prostheses, and all the patients healed properly and had no relapse [143]. Compared to cases of critical long bone defects, the authors of this study managed to use an integrated polymer in metal prostheses in a human body; given the young age of the patients, it can be concluded that this method overcame many drawbacks that were considered great challenges for the future of 3D bioprinting in orthopedics.

To demonstrate the effectiveness of polymers, the behavior of these prostheses in joints must be assessed to adequately evaluate the interaction with the surrounding tissues and to eliminate the risks of bone damage or mechanical failure; these complications are less likely to occur in the case of 3D-printed metallic parts.

We present below a compilation of comprehensive information on the technical features of various biomaterials and the devices used in bioprinting along their physical properties (Table 1).

### 2.4. Fixation Methods of the Prostheses in the Affected Limb and Future Approaches

One of the greatest challenges in replacing an entire bone with a 3D-printed build, besides biocompatibility and ensuring the desired biological activity, is finding a mechanism to anchor the prosthesis to maintain limb functionality. Thus, in the case of a scapular defect resulting in bone replacement, an electron beam melting 3D-printed titanium scapula was stabilized around the patient’s clavicle with a non-absorbable wrap and anchored to the ligaments and bone processes in the small orifices that the prosthesis, as well as the bone, provide [69].

Different parts of the shoulder girdle could be developed using 3D bioprinting as long as a mechanism of fixation is designed, representing a great potential for the replacement of an entire shoulder girdle in the future [144,145]. However, given the complexity of the muscular joint complex of the shoulder girdle, numerous other fixation elements should be added. Current research is centered on the fixation elements of prostheses, with numerous studies proposing holes or anchors where muscles and ligaments can be fixed [146,147,148,149]. However, the prostheses are rigid, have surfaces with high friction, and lack softness [150,151,152]. An optimal fixation needs to extend beyond wire or other materials to just bind the anatomical elements that sustain the bone. Future research should approach different methods to coat the areas of fixation with elastic, resistant, and bioactive materials to ensure an area closer to the biologically active tissue of the body, and this is the case, especially for metal-based prostheses.

**Figure 4:**
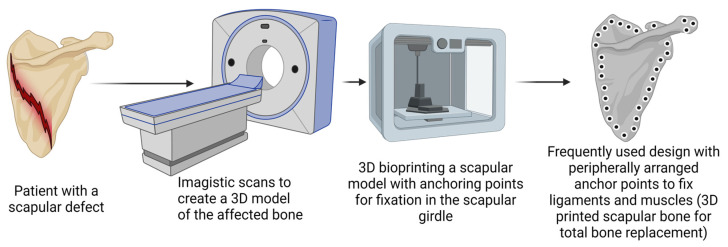
Illustration of a design that could be used for creating anchoring points in total bone prostheses. Created in BioRender. Timofticiuc, I. (2024) BioRender.com/q54g477, date of last access—12 October 2024.

One study managed to overcome the technical challenge of the biocompatible materials used (i.e., the high melting point of tantalum: 2996 °C) and managed to build and place a porous tantalum prosthesis designed with peripheral fixation holes as anchor points for the surrounding tendons and muscles, ensuring the comfort of movement and highly efficient biomechanical mobility [118]. Designing numerous holes in the 3D-printed construct might act as a mechanical destabilizer of the construction; however, they are mandatory for the proper implementation of the prosthesis (Figure 4). Nevertheless, a balance between the dimension of the holes and their frequency in the build needs to be identified to ensure the patients with mobility in all the ranges of motion and no rigidity while not interfering with the mechanical properties of the prosthesis [153,154,155].

For example, the main challenge in saving the functions of the fingers is the fixation of the prostheses at the site of the implantation, and the solution requires the design of anchoring holes and proper positioning to imitate the physiological anchoring points of the tendons and muscles [118,153,154,155]. Studying the fixation methods in other research, we can observe that many holes are generally designed as anchoring points, but ways to eliminate any risk of complications and to reduce the mechanical frictions of metal–bone tissue interaction need to be identified. Also, no prostheses made entirely from biopolymers are yet reported in humans, a situation that could change in the future.

For total replacement of the clavicle with titanium, the fixation in the scapular girdle is more challenging, therefore ensuring that the titanium model has porosities is essential for the fixation with resorbable wire on the clavicle ligaments [69]. Prosthesis fixation can be satisfactory using muscles and tendons as fixation points; however, the extended manufacturing time and expensive production also need to be considered [95].

Bone prostheses can yield great results in different regions of the body when using adequate materials to meet the specific mechanical properties required, but a few challenges still exist and should be approached by future studies such as designing fixation elements and controlling the tendency of these materials to induce osteogenesis and angiogenesis, which may produce long-term defects at the site of the intervention if overamplified.

## 3. Innovations and Future Directions in 3D Bioprinting for Limb Salvage Surgery

With the input of 3D printing in medical activity, the possibility of creating patient-specific constructs increased exponentially [156]. Once the 3D technologies such as extrusion-based 3D bioprinting, inkjet-based 3D bioprinting, laser-assisted 3D bioprinting, and stereolithographic-based 3D bioprinting developed [133], using viable cells in printed scaffolds has introduced transformative innovations in the surgery field by offering the possibility to fabricate biological active parts that mimic the mechanical and physiological characteristics of the native tissues [157]. For example, it was shown that skin can be either 3D bioprinted at the site of the skin defect or bioprinted separately as a scaffold that can be transplanted after postprocessing in a bioreactor. Given the two possibilities, printing in situ was the optimal, cost-effective solution for different skin injuries due to the controlled evolution of the print in time at the site of the trauma [158].

Innovative for limb salvage surgery is the ability to design parts made of biomaterials with properties that enable osteogenesis and angiogenesis. Furthermore, advances in 3D bioprinting allowed the integration of viable cells in special mixtures called bioinks, thus accelerating the reparative events and integration with the native tissue, which was not possible before the 3D printing input [15]. Besides classical 3D printing of silk fibroin, there are mentions of silk also used as a bioink for 3D bioprinting, also known as “silk-inks” [159]. In a study where silk ink was mixed with different enzymes, gold nanoparticles, or various antibiotics, and printed directly in a Petri dish, it was shown that the scaffold is capable of mineralization while protecting itself from bacteria, thus explaining how silk can be a great candidate for use in a human body, but especially for osteogenesis [160].

Although the results are promising, many challenges persist. The incorporation of viable cells is limited within the scaffolds, and many of the cells used are still in experimental stages [161]. Additionally, the biological activity of the constructs is limited to the number of cells included in the scaffold build [162]. Using excessive viable cells in different limb parts can lead to defective mechanical behavior, especially for weight-bearing applications [163].

Current applications of 3D printing in limb salvage surgery are titanium- and polymer-based prostheses [164,165]. Although there are no reported studies where viable cells are directly incorporated in the titanium build, the bioactive properties of a titanium construct were enhanced by coating it with 3D-bioprinted cell-laden hydrogels [166]. Without affecting the surface of the titanium construct, an enhanced interaction with cells and proteins that promotes differentiation and mineralization was established [166]. While the constructs do not include viable cells, they serve as a precursor to bioprinting applications, with the condition of finding the optimal viable cell composition to not interfere with the mechanical structure of the limb parts printed. This highlights the fact that 3D printing usually addresses structural and mechanical needs, with less focus on bioactive needs, while 3D bioprinting mainly addresses the need for biofunctionality in the parts that are created. Combining these approaches could be the bridge to the next era of medical activity, especially in this category of patients [133]. Future directions include refining bioprinting technologies, enhancing the mechanical aspects of fully bioprinted constructs, and also optimizing the design to better support angiogenesis and nerve regeneration, but also to address the costs that these techniques include [133,167,168]. The roadmap to achieving these goals, in our view, is by making hybrid parts that combine the mechanical stability of simple 3D-printed parts with the biological activity of other softer materials such as polymers used also in classical 3D printing and the biological activity of 3D-bioprinted scaffolds. By adapting these technologies, bioprinting can evolve into a comprehensive tool for limb salvage surgery.

## 4. Conclusions

The literature on 3D bioprinting in limb salvage surgery on the lower limb is scarce compared to studies on the upper limb. This might be due to already perfected solutions of prefabricated prostheses for the lower limb; however, upper limb injuries do not benefit from similar solutions. Partial restorative surgeries or bone replacements can be managed with fewer complications compared to the previous decade, leaving room for further development of material characteristics and properties while improving 3D printing techniques and devices.

Many studies show the possibility of creating bioprinted compounds with various viable human cells. Additionally, many studies report various in vitro studies of how these different cells interact with human-like scaffolds. In our view, the innovation and also the solution for the still rapid development of the merged field of 3D printing and medicine is by creating hybrid constructs that can ensure mechanical properties, functional and biofunctional characteristics, and also builds that promote optimal integration with less to no side effects in patients.

Bioprinting overlaps in many ways with classical orthopedic approaches but has the opportunity to provide additional benefits, advantages, and functionality to the builds, showing tremendous potential for the future of limb salvage surgery. The perspective of building constructs besides bioactive bone prostheses that mimic muscles, nerves, and other tissues is one of the reasons for the extensive research conducted in this promising field that holds the potential for identifying the optimal balance for building full-functionality limb prostheses, the ultimate goal of limb salvage surgeries—the possibility of replacing an entire limb, regardless of the disease or trauma that the patients suffer from.

## Figures and Tables

**Table 1:** Characteristics of various bones manufactured with biomaterials using 3D bioprinting.

Body Part	3D Printing Technology	Biocompatible Material	Compatible 3D Printer	Properties and Mentions	Reference
**Bones of the Upper Limb**					
Clavicle	SLA	PEEK	For guiding plate—UP BOX+ 3D printer (Beijing Tiertime Technology Co, Ltd., Beijing, China);	- Low Young’s modulus; - Reduced stress-shielding effect; - Biological strength; - Smooth surface;	[141]
	EBM	Ti	EBM S12 system (Arcam AB, Mölndal, Sweden)	- High Young’s modulus: 110 GPa in comparison with cortical bone: 7–30 GPa; - High stress-shielding effect; - Satisfactory shoulder function and satisfactory ROM: flexion 90°, extension 35°, abduction 80°;	[69]
Scapula	EBM	Ti	EBM S12 system (Arcam AB, Mölndal, Sweden)	- High Young’s modulus: 110 GPa in comparison with cortical bone: 7–30 GPa; - High stress-shielding effect;	[69]
	EBM	Titanium alloy (TiAl_6_V_4_)-Epore	Provided by MUTARS^®^ (Implantcast GmbH, Buxtehude, Germany)	- Porosity: 60%; - Rod thickness: 330–390 µm; - Young’s modulus: 3 GPa - Satisfactory shoulder function and ROM;	[79]
	FFF	PEEK (polyether-ether-ketone)	Surgeon 200, Jugao-AM, Xi’an, China	- von Mises stress: 22.6 MPa; - Layer thickness: 0.2 mm; - Printing speed: 40 mm/s; - Nozzle temperature: 420 °C; - Density: 1.28–1.32 g/cm^3^; - Flexural modulus: 3 GPa; - Flexural strength: 110 MPa; - Thermal conductivity: 0.29 W/m/K^5^.	[140]
Humerus	EBM	Titanium alloy with 6% aluminum and 4% vanadium	Not mentioned	Not mentioned	[95]
	EBM	Titanium alloy (TiAl_6_V_4_)	Chunli^®^, Beijing, China	- Pore size: 500 µm; - Porosity rate: 60%.	[96]
Ulna/Radius	EBM	Titanium alloy with 6% aluminum and 4% vanadium	Not mentioned	Not mentioned	[95]
	EFD-Robocasting	β-Tricalcium Phosphate coated with dipyridamole	EDF Inc., Nordson, Westlake, USA, OH-3D direct-write-micro printer system	- Pore size: 350 µm; - Nozzle diameter: 250 µm; - Dipyramidole coating concentration: 1000 µM;	[40]
	Bioprinting	PLLA (Resomer L-206, Boehringer Ingelheim Pharma KG, Ingelheim, Germany)	3D Bioprinter INVIVO 4D2. ROKIT Healthcare, Seoul, Korea	- Compression strength: 80 ± 10 MPa; - PLLA molecular weight: 100,000 g/mol; - Nozzle diameter: 200 µm; - Extrusion temperature: 200 °C; - Ejection pressure: 200–300 kPa; - Pore size: 480 ± 10 µm; - Grid thickness: 187 ± 2 0 µm; - Porosity: 82%; - Compressive strengths: 100 MPa.	[41]
Carpals, Metacarpals, Phalanges	EBM	Tantalum	UP BOX, Beijing Tiertime Technology Co., Ltd., Beijing, China	- Grip strength 33.4 ± 2.3 kg; - Pinch strength 8.9 ± 0.7 kg; - Flexion: 54.6° ± 0.8°; - Dorsiflexion: 54.7° ± 1.7°; - Ulnar deviation: 34.6° ± 1.9°; - Radial deviation: 25.9° ± 0.8°; - High porosity: 75%; - Low Young’s modulus; - High coefficient of friction; - Thickness: 3.5–4 mm;	[118]
	FFF	PEEK (polyether-ether-ketone)	Apium M220 (Apium Additive Technologies GmbH, Karlsruhe, Germany)	- Nozzle diameter: 0.4 mm; - Density: 1.30 g/cm^3^; - Melting temperature: 340 °C.	[142]
	EBM	Titanium alloy (TiAl_6_V_4_)	Shanghai Shenghsi Medical Devices Co., Ltd.; 3D printer—Arcam A1; Arcam, Mölndal, Sweden	Not mentioned	[119]
	EBM	Titanium	Not mentioned	Not mentioned	[120]
**Bones of the Lower Limb**					
Femur	EBM	Titanium alloy (TiAl_6_V_4_)	Not mentioned	- Pore size: 500 µm; - Porosity: 70%; - Young’s modulus—porous titanium alloy: 1500 MPa; solid titanium alloy: 110,000 MPa; - Poisson’s ratio: 0.30; - Displacement value in the lateral femoral condyle: varied from 0.01–0.07 mm ≈ 0.01 mm to 0.04 mm.	[134]
Tibia and fibula	SLM	Titanium alloy (TiAl_6_V_4_)	Arcam EBM, Gothenburg, Sweden	- Young’s modulus: 1200 ± 48 MPa; - Porosity: 68%; - Pore size: 625 ± 70 µm; - Poisson’s ratio: 0.34;	[137]
	SLM	Titanium alloy (TiAl_6_V_4_) and β-TCP particles added	Xi’an Bright Laser Technology Co., Ltd. (Xi’an, China)	- Pore diameter bioceramic granules: 500–600 µm; - Diameter of the granules: 1.5–3 mm; - Average operation time: 85–385 min; - Young’s modulus: 110 GPa.	[143]

SLA = Stereo-lithography; EBM = Electron beam melting; FFF = Fused Filament Fabrication; SLM = Selective Laser Melting; EFD = Electric Field Driven; PLLA = Poly-L-lactic acid; PEEK = Polyether–Ether–Ketone; Ti = Titanium; β-TCP = β-Tricalcium Phosphate; ROM = Range of Motion.

## Data Availability

No new data were created or analyzed in this study. Data sharing is not applicable to this article.
